# Supplementary material for: Affective state determination in a mouse model of colitis-associated colorectal cancer
Source: PLoS One. 2020 Jan 27;15(1):e0228413. doi: 10.1371/journal.pone.0228413 (PMC6984705; doi:10.1371/journal.pone.0228413)
Supplement: S6 Dataset — (DOCX) [file pone.0228413.s006.docx]

| **Non- Parametric Correlations** | | | | | | | | | | | | | | | | | | |
| --- | --- | --- | --- | --- | --- | --- | --- | --- | --- | --- | --- | --- | --- | --- | --- | --- | --- | --- |
|  | | | grimr19 | grimp19 | burrow19 | dai19 | colscore19 | tumour19 | grimp40 | burrow40 | dai40 | colscore40 | tumour40 | grimp61 | burrow61 | dai61 | colscore61 | tumour61 |
| Spearman's rho | grimr19 | Correlation Coefficient | 1.000 | .163 | -.287 | .631^**^ | .517^**^ | .668^**^ | -.304 | -.251 | .457^**^ | .582^**^ | .714^**^ | .220 | -.230 | .473^**^ | .515^**^ | .744^**^ |
|  |  | Sig. (2-tailed) | . | .327 | .080 | .000 | .001 | .000 | .063 | .129 | .004 | .000 | .000 | .185 | .165 | .003 | .001 | .000 |
|  |  | N | 38 | 38 | 38 | 38 | 38 | 38 | 38 | 38 | 38 | 38 | 38 | 38 | 38 | 38 | 38 | 38 |
|  | grimp19 | Correlation Coefficient | .163 | 1.000 | -.236 | .096 | .240 | .289 | .046 | .009 | .140 | .217 | .278 | -.009 | -.387^*^ | .201 | .246 | .206 |
|  |  | Sig. (2-tailed) | .327 | . | .154 | .564 | .146 | .078 | .785 | .959 | .401 | .190 | .091 | .958 | .016 | .227 | .136 | .214 |
|  |  | N | 38 | 38 | 38 | 38 | 38 | 38 | 38 | 38 | 38 | 38 | 38 | 38 | 38 | 38 | 38 | 38 |
|  | burrow19 | Correlation Coefficient | -.287 | -.236 | 1.000 | -.203 | -.478^**^ | -.405^*^ | .047 | .404^*^ | -.415^**^ | -.370^*^ | -.239 | -.004 | .350^*^ | -.493^**^ | -.414^**^ | -.433^**^ |
|  |  | Sig. (2-tailed) | .080 | .154 | . | .221 | .002 | .012 | .781 | .012 | .010 | .022 | .149 | .982 | .031 | .002 | .010 | .007 |
|  |  | N | 38 | 38 | 38 | 38 | 38 | 38 | 38 | 38 | 38 | 38 | 38 | 38 | 38 | 38 | 38 | 38 |
|  | dai19 | Correlation Coefficient | .631^**^ | .096 | -.203 | 1.000 | .689^**^ | .764^**^ | -.032 | -.479^**^ | .639^**^ | .658^**^ | .768^**^ | .121 | -.157 | .516^**^ | .610^**^ | .745^**^ |
|  |  | Sig. (2-tailed) | .000 | .564 | .221 | . | .000 | .000 | .848 | .002 | .000 | .000 | .000 | .469 | .348 | .001 | .000 | .000 |
|  |  | N | 38 | 38 | 38 | 38 | 38 | 38 | 38 | 38 | 38 | 38 | 38 | 38 | 38 | 38 | 38 | 38 |
|  | colscore19 | Correlation Coefficient | .517^**^ | .240 | -.478^**^ | .689^**^ | 1.000 | .887^**^ | -.085 | -.512^**^ | .779^**^ | .818^**^ | .794^**^ | .009 | -.238 | .885^**^ | .785^**^ | .829^**^ |
|  |  | Sig. (2-tailed) | .001 | .146 | .002 | .000 | . | .000 | .612 | .001 | .000 | .000 | .000 | .960 | .151 | .000 | .000 | .000 |
|  |  | N | 38 | 38 | 38 | 38 | 38 | 38 | 38 | 38 | 38 | 38 | 38 | 38 | 38 | 38 | 38 | 38 |
|  | tumour19 | Correlation Coefficient | .668^**^ | .289 | -.405^*^ | .764^**^ | .887^**^ | 1.000 | -.193 | -.492^**^ | .797^**^ | .857^**^ | .857^**^ | .016 | -.278 | .861^**^ | .857^**^ | .947^**^ |
|  |  | Sig. (2-tailed) | .000 | .078 | .012 | .000 | .000 | . | .247 | .002 | .000 | .000 | .000 | .925 | .092 | .000 | .000 | .000 |
|  |  | N | 38 | 38 | 38 | 38 | 38 | 38 | 38 | 38 | 38 | 38 | 38 | 38 | 38 | 38 | 38 | 38 |
|  | grimp40 | Correlation Coefficient | -.304 | .046 | .047 | -.032 | -.085 | -.193 | 1.000 | -.090 | -.243 | -.147 | -.194 | -.108 | -.065 | -.158 | -.080 | -.188 |
|  |  | Sig. (2-tailed) | .063 | .785 | .781 | .848 | .612 | .247 | . | .590 | .142 | .379 | .244 | .520 | .699 | .344 | .631 | .258 |
|  |  | N | 38 | 38 | 38 | 38 | 38 | 38 | 38 | 38 | 38 | 38 | 38 | 38 | 38 | 38 | 38 | 38 |
|  | burrow40 | Correlation Coefficient | -.251 | .009 | .404^*^ | -.479^**^ | -.512^**^ | -.492^**^ | -.090 | 1.000 | -.465^**^ | -.393^*^ | -.408^*^ | .333^*^ | .373^*^ | -.504^**^ | -.452^**^ | -.532^**^ |
|  |  | Sig. (2-tailed) | .129 | .959 | .012 | .002 | .001 | .002 | .590 | . | .003 | .015 | .011 | .041 | .021 | .001 | .004 | .001 |
|  |  | N | 38 | 38 | 38 | 38 | 38 | 38 | 38 | 38 | 38 | 38 | 38 | 38 | 38 | 38 | 38 | 38 |
|  | dai40 | Correlation Coefficient | .457^**^ | .140 | -.415^**^ | .639^**^ | .779^**^ | .797^**^ | -.243 | -.465^**^ | 1.000 | .751^**^ | .650^**^ | -.013 | -.290 | .791^**^ | .840^**^ | .757^**^ |
|  |  | Sig. (2-tailed) | .004 | .401 | .010 | .000 | .000 | .000 | .142 | .003 | . | .000 | .000 | .938 | .078 | .000 | .000 | .000 |
|  |  | N | 38 | 38 | 38 | 38 | 38 | 38 | 38 | 38 | 38 | 38 | 38 | 38 | 38 | 38 | 38 | 38 |
|  | colscore40 | Correlation Coefficient | .582^**^ | .217 | -.370^*^ | .658^**^ | .818^**^ | .857^**^ | -.147 | -.393^*^ | .751^**^ | 1.000 | .770^**^ | .129 | -.326^*^ | .868^**^ | .835^**^ | .828^**^ |
|  |  | Sig. (2-tailed) | .000 | .190 | .022 | .000 | .000 | .000 | .379 | .015 | .000 | . | .000 | .440 | .046 | .000 | .000 | .000 |
|  |  | N | 38 | 38 | 38 | 38 | 38 | 38 | 38 | 38 | 38 | 38 | 38 | 38 | 38 | 38 | 38 | 38 |
|  | tumour40 | Correlation Coefficient | .714^**^ | .278 | -.239 | .768^**^ | .794^**^ | .857^**^ | -.194 | -.408^*^ | .650^**^ | .770^**^ | 1.000 | .125 | -.263 | .672^**^ | .700^**^ | .823^**^ |
|  |  | Sig. (2-tailed) | .000 | .091 | .149 | .000 | .000 | .000 | .244 | .011 | .000 | .000 | . | .455 | .111 | .000 | .000 | .000 |
|  |  | N | 38 | 38 | 38 | 38 | 38 | 38 | 38 | 38 | 38 | 38 | 38 | 38 | 38 | 38 | 38 | 38 |
|  | grimp61 | Correlation Coefficient | .220 | -.009 | -.004 | .121 | .009 | .016 | -.108 | .333^*^ | -.013 | .129 | .125 | 1.000 | -.032 | -.051 | .080 | -.027 |
|  |  | Sig. (2-tailed) | .185 | .958 | .982 | .469 | .960 | .925 | .520 | .041 | .938 | .440 | .455 | . | .850 | .763 | .633 | .872 |
|  |  | N | 38 | 38 | 38 | 38 | 38 | 38 | 38 | 38 | 38 | 38 | 38 | 38 | 38 | 38 | 38 | 38 |
|  | burrow61 | Correlation Coefficient | -.230 | -.387^*^ | .350^*^ | -.157 | -.238 | -.278 | -.065 | .373^*^ | -.290 | -.326^*^ | -.263 | -.032 | 1.000 | -.321^*^ | -.538^**^ | -.266 |
|  |  | Sig. (2-tailed) | .165 | .016 | .031 | .348 | .151 | .092 | .699 | .021 | .078 | .046 | .111 | .850 | . | .049 | .000 | .106 |
|  |  | N | 38 | 38 | 38 | 38 | 38 | 38 | 38 | 38 | 38 | 38 | 38 | 38 | 38 | 38 | 38 | 38 |
|  | dai61 | Correlation Coefficient | .473^**^ | .201 | -.493^**^ | .516^**^ | .885^**^ | .861^**^ | -.158 | -.504^**^ | .791^**^ | .868^**^ | .672^**^ | -.051 | -.321^*^ | 1.000 | .863^**^ | .838^**^ |
|  |  | Sig. (2-tailed) | .003 | .227 | .002 | .001 | .000 | .000 | .344 | .001 | .000 | .000 | .000 | .763 | .049 | . | .000 | .000 |
|  |  | N | 38 | 38 | 38 | 38 | 38 | 38 | 38 | 38 | 38 | 38 | 38 | 38 | 38 | 38 | 38 | 38 |
|  | colscore61 | Correlation Coefficient | .515^**^ | .246 | -.414^**^ | .610^**^ | .785^**^ | .857^**^ | -.080 | -.452^**^ | .840^**^ | .835^**^ | .700^**^ | .080 | -.538^**^ | .863^**^ | 1.000 | .827^**^ |
|  |  | Sig. (2-tailed) | .001 | .136 | .010 | .000 | .000 | .000 | .631 | .004 | .000 | .000 | .000 | .633 | .000 | .000 | . | .000 |
|  |  | N | 38 | 38 | 38 | 38 | 38 | 38 | 38 | 38 | 38 | 38 | 38 | 38 | 38 | 38 | 38 | 38 |
|  | tumour61 | Correlation Coefficient | .744^**^ | .206 | -.433^**^ | .745^**^ | .829^**^ | .947^**^ | -.188 | -.532^**^ | .757^**^ | .828^**^ | .823^**^ | -.027 | -.266 | .838^**^ | .827^**^ | 1.000 |
|  |  | Sig. (2-tailed) | .000 | .214 | .007 | .000 | .000 | .000 | .258 | .001 | .000 | .000 | .000 | .872 | .106 | .000 | .000 | . |
|  |  | N | 38 | 38 | 38 | 38 | 38 | 38 | 38 | 38 | 38 | 38 | 38 | 38 | 38 | 38 | 38 | 38 |
| **. Correlation is significant at the 0.01 level (2-tailed). | | | | | | | | | | | | | | | | | | |
| *. Correlation is significant at the 0.05 level (2-tailed). | | | | | | | | | | | | | | | | | | |
