# Supplementary material for: Affective state determination in a mouse model of colitis-associated colorectal cancer
Source: PLoS One. 2020 Jan 27;15(1):e0228413. doi: 10.1371/journal.pone.0228413 (PMC6984705; doi:10.1371/journal.pone.0228413)
Supplement: Attachment — Submitted filename: Response to Reviewers.docx [file pone.0228413.s008.docx]

**Responses to Reviewers – 13^th^ January 2020**

**Title:** Affective state determination in a mouse model of colitis-associated colorectal cancer

**Authors:** Lauren C Chartier, Michelle L Hebart, Gordon S Howarth, Alexandra L Whittaker and Suzanne Mashtoub

**Manuscript ID:** PONE-D-19-22786

The authors would kindly like to thank the two reviewers for their comments and questions regarding the current manuscript. All answers to comments and revisions have been documented in the manuscript file labelled ‘Revised Manuscript with Track Changes’. Page/line number references for amendments are listed in this document for your convenience. Additionally, a copy of the completed reviewed manuscript has been uploaded with the revision and is labelled ‘Manuscript’.

**Reviewer #1:** *All comments have been satisfactory and thoroughly addressed. I recommend the current manuscript for publication in PLOS ONE.*

**Reviewer #2:** *I thank the authors for their response to my questions and comments. However, there are still a few points that have not been addressed and revised in the manuscript:

- Although you mentioned that “mice were stratified to groups based on baseline body weight and burrowing activity” (P6LL128-130), there were significant differences in burrowing activity at baseline and you stated in the discussion part that “in future studies, it would be beneficial to allocate treatment groups based on the burrowing activity”. Please check the statement given in the part “experimental design” in LL128-130.*

It was originally stated that bodyweight and burrowing ability was used to assign groups as mice were firstly stratified to treatment groups based on their baseline bodyweight, then baseline burrowing data was used to ensure that burrowing ability was equally distributed across groups. The significant differences in baseline burrowing ability was unavoidable due to control groups being utilised in another study of the same nature running at the same time, as stated in the ‘animal studies’ section of the Material and Methods. However, to avoid confusion for the reader, the authors have revised the ‘experimental design’ section and now reads ‘Mice were stratified to groups based on baseline body weight.’ (P6L129). The statement in the discussion remains as “in future studies, it would be beneficial to allocate treatment groups based on the burrowing activity”. *- Please provide information on the health status of the mice (see ARRIVE guidelines).*

The health status of mice has now been included in the ‘Animal Studies’ section of the Methods. The included statement (P6L117-119) reads; ‘The ARC undertakes a quarterly health screening, covering various bacterial, viral and parasitic organisms, all of which the obtained colony screened negative for.’ *- Material and Methods, DAI (P8LL157-162): Please indicate that scorer(s) were not blinded and that DAI scores were obtained before administration of buprenorphine.*

We have now included that scorers were not blinded to treatment groups when obtaining DAI measurements and that DAI was calculated prior to buprenorphine administration (P7L153-P8L160).  *- Material and Methods, MGS: Time of day was not added to this section. Moreover, the authors described that there was one MGS scorer only, but in the response to my comments three scorers are listed (Alexandra Whittaker, Rebecca George, and Lauren Chartier). Did each of them score a third of the mouse faces? If this is true please add this information to the MGS methods section.*

In the MGS section of the experimental design, we stated that MGS scoring occurred in the morning following buprenorphine administration; however, to clarify this we have now included a time (approximately 9am-12pm) on P9L215. There was only one MGS scorer per mouse, however, due to the large number of animals and time restraints, live scoring was shared amongst the three grimace experienced researchers (Alexandra Whittaker, Rebecca George, and Lauren Chartier), whereby one researcher scored one mouse. Retrospective blinded scoring was all performed by Miss Lauren Chartier.  *- The authors stated in their responses: “Mice were administered thrice weekly with buprenorphine. These administrations occurred in the morning, 2 hours prior to MGS scoring and 8 hours prior to burrowing analyses.”, “Furthermore, live MGS scores/videos for retrospective scoring were obtained in the morning (9-12pm; 2 hours following
buprenorphine administration)”. I assume that PM is a typing error (AM?) and DAI was obtained at 9 AM, buprenorphine was administered at 9 AM, mouse faces were scored at noon (MGS) and burrowing was monitored at 6 PM. According to this schedule, DAI could not asses the analgesic effects of buprenorphine (in contrast to the MGS and the burrowing test). Therefore, I would recommend emphasizing this issue and make it clearer for the reader in the discussion (especially in P20LL335-346; in this passage the reader has the impression that DAI was considered to assess the analgesic effect of buprenorphine administered on the respective days). Due to the short lasting analgesic effects of buprenorphine, it is unlikely that you have “picked up the effect of buprenorphine in the clinical scoring analysis”, as you also stated in your response, but of course you may have measured side effects.*

To clarify the timeline of events for analyses for all mice - DAI was measured first thing in the morning (7-8am) prior to buprenorphine administration (9am). Then MGS scoring/video recordings occurred between 9am-12pm, and finally burrowing analyses were performed from 6pm on selected days. We have included the approximate time of each analysis in the ‘Materials and Methods’ section of the manuscript as well as time following buprenorphine administration to avoid confusion. It is correct that at this timeline we were unlikely to pick up the analgesic effect of buprenorphine in DAI as the effects would not have lasted 24 hours, however this has been stated in the methods that DAI was measured prior to administration (P7L156). Furthermore, MGS was performed at a time where the effects of buprenorphine should have been measureable.

*Moreover, results of the burrowing test should be interpreted with more caution in the discussion part as baseline scores were significantly different – this makes it very tricky and should be pointed out for the reader.*

We have now added to the final paragraph of the discussion (P22L387-388) to highlight that burrowing was significantly different at baseline in AOM/DSS controls and therefore may have impacted the results obtained at other time-points within the study.

*The statement “Overall, results were unable to conclude a significant impact of opioid analgesic (buprenorphine) intervention on the measures used, highlighted by the minimal differences in grimace scores, burrowing behaviour and DAI in disease mice.” (P20LL340-343) does not consider, 1) that DAI also includes pain-specific behaviors and was actually increased in AOM+DSS groups, 2) that the time of DAI scoring did not allow for the assessment of analgesic effects of buprenorphine, 3) and that burrowing data are difficult to interpret due to baseline differences. Based on the difficulties stated above, I would also recommend rethinking the three hypotheses given in LL343-346.*

1) P20L326 in the discussion section has been reworded to highlight that buprenorphine did not have an effect in terms of reducing pain (as this was the purpose of administering analgesia) in AOM/DSS mice, as buprenorphine did impact DAI scores at some time-points.
2) In the current study, DAI was measured daily in the morning prior to buprenorphine administration. This time-point was selected as DAI was routinely incorporated into the daily monitoring of all mice in the study. Furthermore, the effect of buprenorphine on DAI parameters (including bodyweight loss and diarrhoea) did not confound the study as these are retrospective and slow pathological changes (P22L380-382). For example, if DAI was measured 30 mins follow buprenorphine administration, changes in bodyweight and stool consistency would not be identifiable in this short time-frame.
3) The authors acknowledge that the discrepancies in baseline burrowing data mean that these results are difficult to interpret. This issue has been identified in the discussion (P22L390-392) and that future studies should aim to have groups with relatively similar baseline burrowing ability where possible. However, based on the burrowing results from other time-points in the study it is likely that buprenorphine does not have an effect on burrowing behaviour.

In light of these responses, the authors still believe that the hypothesis mentioned on P19L328-331 of the discussion are reasonable.

*Moreover, the following phrase needs to be reformulated with regard to the above-mentioned concerns: “Hence, taken together the results suggest that buprenorphine is ineffective in improving wellbeing in mice with colitis associated colorectal cancer.” (P22LL388-390)*

We agree that the statement highlighted is a little too strong and have modified it. The statement now reads ‘Hence, these data cannot confirm an action of buprenorphine in reducing pain based on the MGS scores obtained, nor any improvement in wellbeing based on DAI score or burrowing behaviour. However, this needs to be considered in light of the difficulty in teasing apart beneficial, versus side effects using the DAI, and the differences obtained in baseline burrowing score. (P21L378-383).

Furthermore, earlier in the same paragraph we have added more clarification that the timing of DAI measurements in respect to buprenorphine-administration is likely why minimal effects were observed (P21L367-368).
